# Mechanotransduction through adhesion molecules: Emerging roles in regulating the stem cell niche

**DOI:** 10.3389/fcell.2022.966662

**Published:** 2022-09-12

**Authors:** Ryan Lim, Avinanda Banerjee, Ritusree Biswas, Anana Nandakumar Chari, Srikala Raghavan

**Affiliations:** ^1^ A∗STAR Skin Research Lab (ASRL), Agency for Science, Technology and Research (A*STAR) 8A Biomedical Grove, Singapore, Singapore; ^2^ Institute for Stem Cell Science and Regenerative Medicine (inStem), GKVK Campus, Bangalore, India; ^3^ Sastra University, Thanjavur, TN, India

**Keywords:** mechanotransduction, stem cell niche, adhesion molecules, adherens junctions, focal adhesion, disease and regenerative therapeutics

## Abstract

Stem cells have been shown to play an important role in regenerative medicine due to their proliferative and differentiation potential. The challenge, however, lies in regulating and controlling their potential for this purpose. Stem cells are regulated by growth factors as well as an array of biochemical and mechanical signals. While the role of biochemical signals and growth factors in regulating stem cell homeostasis is well explored, the role of mechanical signals has only just started to be investigated. Stem cells interact with their niche or to other stem cells via adhesion molecules that eventually transduce mechanical cues to maintain their homeostatic function. Here, we present a comprehensive review on our current understanding of the influence of the forces perceived by cell adhesion molecules on the regulation of stem cells. Additionally, we provide insights on how this deeper understanding of mechanobiology of stem cells has translated toward therapeutics.

## 1 Introduction

Regenerative Medicine, which deals with the restoration of tissues/organs upon injury or chronic disease, is a promising and emerging branch of medical research (Mason and Dunnill, 2008; Mahla, 2016). It is well known that stem cells in our body hold the capability of self-renewal and differentiation into other types of cells, which makes it an ideal candidate for regenerative medicine. Though the term stem cell (original: ‘Stammzelle’) appeared in the scientific literature in the mid-19th century by famous German biologist Ernst Haeckel (Haeckel, 1868); major breakthroughs in the stem cell research only happened a century later. However, over the past 20 years, there have been immense advancements in the field of stem cell research, in elucidating their characteristics and potential for regenerative medicine (Mahla, 2016; Liu et al., 2020).

Although there are different types of stem cells, they all share common characteristics of self-renewal and the ability to differentiate into different cell types. Among many types of stem cell classifications, two common ways to classify them are 1) based on their origin or residency, known as tissue-specific stem cells and 2) based on their potency, which refers to their ability to differentiate into various cell types.

Stem cells isolated from mammalian blastocysts are also known as embryonic stem cells (ESCs) (Evans and Kaufman, 1981; Thomson, 1998; Smith, 2001). Stem cells isolated from the epiblast stage of embryos are termed epiblast stem cells (EpiSCs) and primordial germ cells (embryogenic germ cells EGCs) (Shamblott et al., 1998; Yu and Thomson, 2008). In contrast to ESCs, adult stem cells are found in various tissues and are alternatively termed as somatic stem cells (Leblond, 1964; Robey, 2000). Hematopoietic stem cells (HSC) were amongst the first identified adult stem cells and the most widely studied (Seita & Weissman, 2010). Some of the tissues where pools of adult stem cells have been identified include 1) The nervous system which includes parts of brain, 2) Bone marrow and blood which consist of bone marrow, peripheral blood, dental pulp and spinal cord, blood vessels, 3) Endothelial Progenitor Cells, 4) Skeletal Muscle Stem Cells, 5) Epithelial Cell Precursors in the Skin and Digestive System as well as from parts of cornea, retina, liver, and pancreas. These tissue specific stem cells (TSSCs) behave differently depending on their local environment and the homeostatic requirement of the body.

Stem cells (SCs) often reside in tissue-specific microenvironments termed as the stem cell niche, that play an important role in regulating the proliferation and differentiation of these cells, thereby maintain tissue homeostasis (Schofield, 1978). The concept and significance of the stem cell niche was first proposed by Scholfield in 1978, when he demonstrated that removing these cells from their niche resulted in their differentiation (Schofield, 1978). Stem cell fates are regulated by both intrinsic and extrinsic factors. Cell-intrinsic factors include specific transcription factors, microRNAs, epigenetic regulators and secreted signaling molecules such as Wnts and BMPs (Watt and Hogan, 2000; Nusse, 2008; Gangaraju and Lin, 2009; Wang and Wagers, 2011; Li et al., 2017; Chacón-Martínez et al., 2018; Rigillo et al., 2021). Extrinsic factors include the regulation of stem cells with through cell-cell and cell-substratum interactions (Watt and Huck, 2013; Gattazzo et al., 2014). In addition to the above-mentioned niche factors, mechanical signals originating from cell-intrinsic and externally applied forces also have major implications on the behavior of the SCs (Vining and Mooney, 2017). Force generation by actomyosin contractility and cytoskeletal assembly impinge on mechanisms by which the cells exert intrinsic forces on its extracellular milieu and neighboring cells. In contrast, cell-extrinsic forces arise from the rigidity, topography and composition of the ECM that exerts shear, tensile and compressive forces on the stem cells (Lo et al., 2000; Even-Ram et al., 2006; Dupont et al., 2011; Li et al., 2011; Wolfenson et al., 2019). Transmission of these mechanical forces through cell-cell junctions and cell-substratum adhesions plays a key role in regulating intracellular signaling pathways, eventually determining stem cell fate (Vining and Mooney, 2017). Although the role of cell-intrinsic factors and signaling molecules regulating the quiescence of the stem cells have been extensively studied, the mechanical function of the cell-cell adhesion molecules in maintaining the stem cell homeostasis is less well explored. Cells typically sense and transmit external forces to specialized structures at the cell periphery. These structures then transduce the forces into a biochemically detectable signal, and ultimately the response of the cell is transcribed by the nucleus. These are commonly referred to as mechanotransmission, mechanosensing, and mechanoresponse, respectively or as a whole, mechanotransduction (Ingber, 2006; Jansen et al., 2017). Recent studies have highlighted the importance of biophysical attributes of the microenvironment, including mechanical loading and substrate material property, in determining the self-renewal and commitment of SCs required for its maintenance and differentiation respectively. External forces applied to the SCs from its niche generates traction forces as well as compressive forces through the cytoskeleton. These forces are then transmitted from the cytoskeleton to other cellular components inside or outside of the SCs. This concept of organizing and stabilizing the cytoskeleton by the living cells in response to applied external forces is based on the model of tensegrity, which was first described by D. Ingber (Chen and Ingber, 1999; Ingber, 2003). The cellular tensegrity model describes the cell as a mechanical force bearing structure that is composed of compression resistant components and tension bearing cables. These cables create a pre-stress state in the cell, which is opposed by the compression resistant components known as struts, that aids in the maintenance of the mechanical equilibrium of the cell. This tensegrity-based model highlights the integration of cytoplasmic mechanotransduction and biological responses that are critical for determining the proliferative and differentiative characteristics of SCs, gene expression, as well as its role in organ development (Chowdhury et al., 2010; Engler et al., 2004; 2006). The 2 cell junctions that regulate mechanotransduction of the SCs within its niche are, the cadherin mediated Adherens Junctions (AJs) and the integrin mediated Focal Adhesions (FAs).

## 2 Role of extracellular matrix molecule and adhesion molecules in regulating mechanotransduction of stem cell niche

### 2.1 Extracellular matrix and matrix metalloproteinases as a niche factor

#### 2.1.1 Extracellular matrix

**FIGURE 1:**
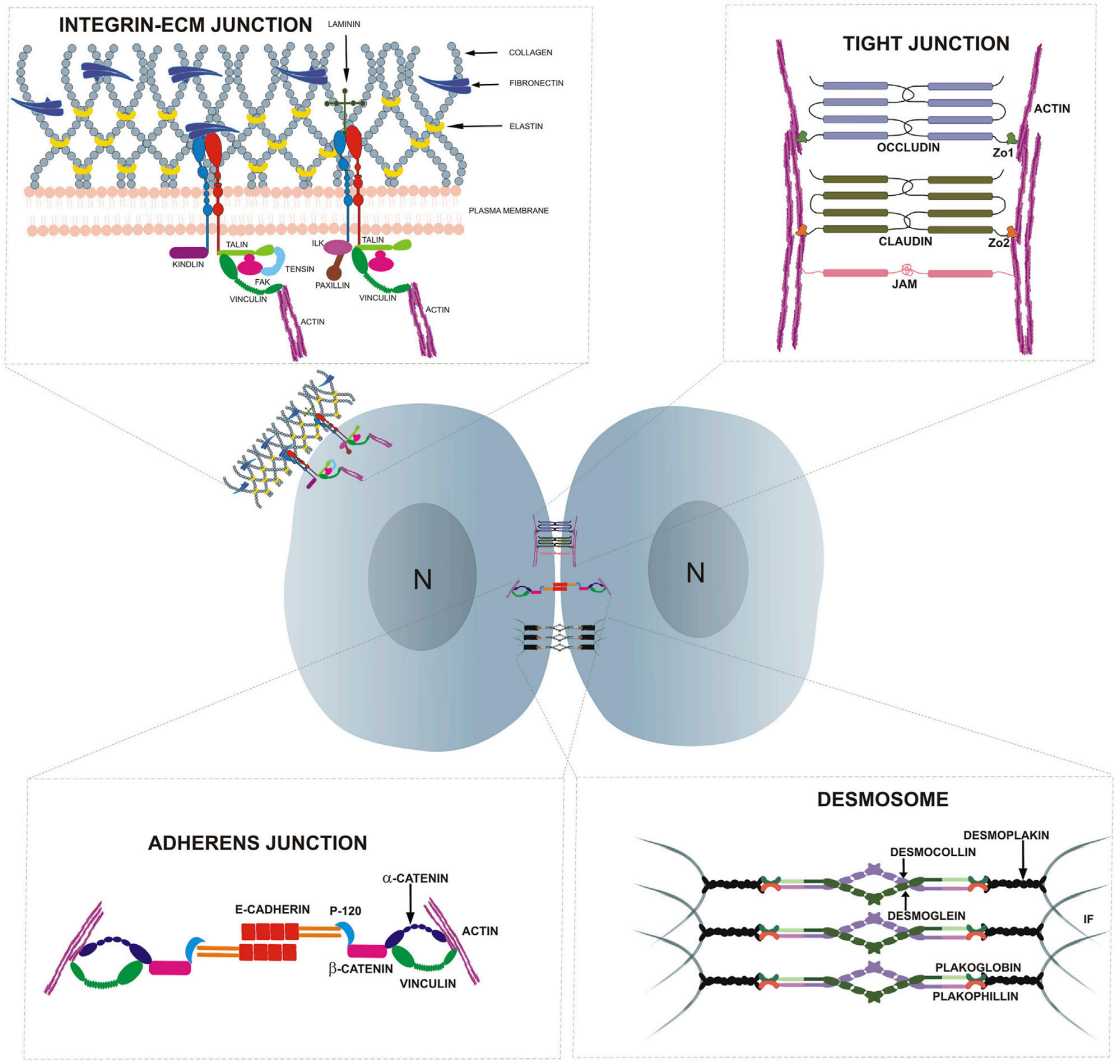
Highlights the cell adhesion molecules present at cell-cell and cell-substratum junctions in epithelial cells.

The ECM comprises of noncellular components surrounding all the cells in the body, and provides mechanical support via a structural macromolecular network (Murphy-Ullrich & Sage, 2014; Theocharis et al., 2016; Stanton et al., 2019). Mechanical cues from the ECM help to regulate cell phenotypes, motility, biochemistry and matrix production and therefore plays an essential role in tissue remodeling (Jaalouk and Lammerding, 2009; Akhmanova et al., 2015). The components of the ECM that mediate signaling in cells include: collagen, elastin, laminin, fibronectin, hyaluronic acid, chondroitin sulfate and syndecans, as well as soluble components such as cytokines, matrix metalloproteinases, growth factors and proteases (Figure 1) (Hynes, 2009; Mohammed et al., 2019). The physical properties of the ECM such as its rigidity, viscosity, porosity and topography are influenced by the concentration, type, and arrangement of macromolecules (fibers, proteoglycans and glycoproteins). As a result, the ECM may present as a soft or hard substrate (Yue, 2014; Jansen et al., 2015). Multiple studies have suggested the role of ECM stiffness as a major regulatory physical factor in determining stem cell fate (Discher et al., 2005; Paszek et al., 2005; Engler et al., 2006; Even-Ram et al., 2006; Hamidouche et al., 2009; Li et al., 2011; Gandavarapu et al., 2014).

##### 2.1.1.1 Collagen

About 30% of the total protein mass in mammals is constituted by collagen, which is also one of the most abundant components of the ECM (Shoulders and Raines, 2009; Ricard-Blum, 2011). The collagen super family comprises 28 members with at least 46 distinct polypeptide chains in vertebrates (Brinckmann, 2005). The primary component of the ECM in vertebrates is collagen I, which consists of stiff, thick and long fibrils that contribute to the structural architecture, shape and mechanical properties of tissues such as tensile strength in skin and resistance to traction in ligaments (Ricard-Blum, 2011). Different collagen types are reported to perform tissue-specific functions. For example, collagen XXII which is present only at tissue junctions (skeletal and heart muscles) and in the skin epithelia acts as an adhesion ligand (Koch et al., 2004). Collagen XIII in bone regulates its formation by coupling bone mass to mechanical use (Ylönen et al., 2005) and collagen VII in skin is critical for maintaining dermal-epidermal adhesion, thus affecting its integrity (Bruckner-Tuderman et al., 1987).

Collagen in the ECM of the stem cell niche plays an important role in determining the proliferative and differentiation potential of the stem cells. Studies have shown that type IV collagen in the basement membrane and hair follicles of the skin provides mechanical support as well as an increase in the proliferative potential of normal human basal keratinocytes (Breitkreutz et al., 2013; Choi et al., 2014). Likewise, in muscle stem cells (satellite cells), alterations of muscle stiffness which is mediated by collagen VI deposition by fibroblasts affects the self-renewal and maintenance of these stem cells (Urciuolo et al., 2013). The mechanical microenvironment of the intestinal crypt cells has been shown to be primarily regulated by type VI collagen via its interaction with the RGD domain of fibronectin (Groulx et al., 2011; Benoit et al., 2012). Altered tissue stiffness in the intestinal crypt cells is associated with increased type VI collagen secretion into the basal lamina. This alteration of the tissue stiffness, in turn, affects integrin focal adhesions, growth factor receptor signaling and acto-myosin and cytoskeletal-dependent cellular contractility (Handorf et al., 2015). Collagen has also been shown to support stem cell properties such as clonogenicity, cell growth and osteogenic differentiation potential (Chen et al., 2007; Linsley et al., 2013; Somaiah et al., 2015; Akhir and Teoh, 2020). The absence of collagen III in mice affects osteoblast differentiation (Volk et al., 2014) whereas the denaturation of collagen I promotes osteoblastic differentiation (Tsai et al., 2010). Taken together, these studies suggested that the structural integrity conferred by collagen in the ECM is implicated in the fate of stem cell proliferation and differentiation in different niches.

##### 2.1.1.2 Elastin

Another important component factor of the ECM along with collagen are the elastin networks that are predominantly found in soft elastic tissues like the skin, blood vessels and lungs (Rosenbloom et al., 1993; Li & Daggett, 2002). Elastin is formed from its monomer, tropoelastin. Tropoelastin is crosslinked at its lysine residues, thereby forming elastic fibers along with microfibrillar proteins such as fibrillin. These elastic fibers are the major contributors to the mechanical properties of elastin (Aaron and Gosline, 1981; Ozsvar et al., 2015). Elastin is predominantly deposited during prenatal development and childhood and is rarely synthesized during adulthood (Ozsvar et al., 2015). 90% of elastin comprises elastic fibers, with the remaining 10% consisting of fibrillin glycoprotein (Mecham, 1991). The primary role of these elastic fibers is to undergo constant stretch-recoil cycles, thus maintaining the structural and functional integrity of the elastic tissues throughout the organism’s lifetime (Ozsvar et al., 2015).

Studies have shown that tropoelastin can promote cell attachment and migration of various cell types such as fibroblasts, mesenchymal stem cells (MSCs) and endothelial cells (Ozsvar et al., 2015). Additionally, tropoelastin has been shown to interact and bind with one of the major cell adhesion proteins, integrins, to promote proliferation in MSCs and HSCs. Specifically, elastin’s interaction with ɑvβ3 and ɑvβ5 integrins on the surface of MSCs, either as surface-bound or as soluble tropoelastin, promotes MSC proliferation. This was attributed to the ability of elastin to convey mechanical signals and modulate gene expression that supports proliferation (Rodgers and Weiss, 2004; Yeo and Weiss, 2019). Tropoelastin-integrin interactions partially contribute to the wound healing process by remodeling the ECM (Ozsvar et al., 2021). Likewise, in the hematopoietic stem cell (HSC) compartment, mechanical characteristics of tropoelastin in the ECM has been shown to be associated with its differentiation (Holst et al., 2010). The tunable elasticity of elastin plays an important role in regulating cell proliferation, differentiation, adhesion and migration.

##### 2.1.1.3 Laminin

Laminins are a family of large multidomain, heterotrimeric glycoproteins that are composed of three different subunits, the α, β, and γ chains. These chains are encoded by 11 genes in humans and form at least 15 trimeric combinations of subunits throughout different tissues (Aumailley et al., 2005). Different combinations of these three subunits confer upon laminins its nomenclature. For example, laminin composed of α2, β1, and γ1 chains is laminin 211 and so on (Kaur and Reinhardt, 2015). Each laminin subunit consists of tandem LE repeats (laminin-type epidermal growth factor-like domains), globular domains interspersed within the LE domains and an α-helical domain which follows after the LE repeats (Beck et al., 1990; Hamill et al., 2009). The α-helical domains of the α, β, and γ subunits wind around each other to produce a trimeric coiled-coil structure (Beck et al., 1990). Laminins (with other components) maintain the structural and functional integrity of the ECM, ultimately affecting cell viability (Armony et al., 2016). Additionally, laminins also influence cell adhesion and migration (Domogatskaya et al., 2012).

Laminins are well known for regulating different stem cell functions such as cell-substratum adhesion, proliferation, migration and differentiation (Iorio et al., 2015). However, there are very few *in-vivo* reports that highlight the mechanical properties of laminin in maintaining stem cell function. In contrast, *in-vitro* studies that modulate substrate stiffness and ligand composition to mimic the native elasticity of the stem cell niches have highlighted the role of laminin as a stem cell niche factor. For example, satellite cell renewal from skeletal muscle was promoted when cultured on laminin crosslinked hydrogels (with a stiffness of ∼12 kPa) which mimics the native elasticity of skeletal muscles. This study further reported that muscle satellite cells cultured on laminin functionalized hydrogels contributed to muscle regeneration when grafted into immune deficient mice that partially lacked endogenous satellite cells (Gilbert et al., 2010). Different isoforms of laminin have been shown to be able to direct the differentiation of human induced pluripotent stem cells (hiPSCs) into multiple eye-like tissues. Of note, the E8 fragments of laminin 511 enables the dense concentration of hiPSCs colonies due to actomyosin contraction, which results in cell density YAP inactivation and ultimately leading to retinal differentiation in these colony centers (Shibata et al., 2018). Neural stem and progenitor cells (NSPCs) differentiation towards the oligodendrocyte lineage have been shown to be favored when cultured on laminin coated substrate over fibronectin coated substrate and along with mechanical stimulation by subjecting the cells to a single static stretch (Arulmoli et al., 2015). This oligodendrocyte lineage specific differentiation is preferentially mediated by the integrin α6 laminin binding. These findings correlate with what is known *in vivo* as laminin is found in high concentrations in a developing brain and is also expressed in the NSPC niches such as the subventricular zone (SVZ) of the lateral ventricles and dentate gyrus (DG) of the hippocampus in an adult brain (Chen et al., 2003; Lathia et al., 2007; Relucio et al., 2012). Thus, mechanical forces resulting from robust cell movements during development and tissue folding of the brain may thus be implicated together with laminin in the ECM to direct NSPC differentiation towards the oligodendrocyte lineage within this niche.

##### 2.1.1.4 Fibronectin

Fibronectin (FN) is a large 440 kDa, multidomain, dimeric glycoprotein which is found in the extracellular milieu of different tissues (Proctor, 1987; Parisi et al., 2020). It is composed of two identical subunits that are joined together by disulfide bonds near the C-terminal regions. Each subunit monomer consists of three different types of repeats, namely FNI, FNII and FNIII (Hynes, 1986; Pankov and Yamada, 2002; To and Midwood, 2011; Adams et al., 2015). FN exists in both soluble and insoluble forms. The soluble FN, also known as plasma fibronectin, circulates in the blood and contributes primarily to tissue repair by forming fibrin blood clots (To and Midwood, 2011). On the other hand, the insoluble FN, also known as cellular FN, is found on cell surfaces and in the ECM (Proctor, 1987; To and Midwood, 2011). FN is also considered as the ‘master organizer’ of the ECM since it helps in organizing other matrix proteins such as collagen, heparin, fibrillin and fibulins (Morla and Ruoslahti, 1992; Chung and Erickson, 1997; Klass et al., 2000; Woods, 2001; Galante and Schwarzbauer, 2007; Kaur and Reinhardt, 2015). The tripeptide sequence Arg-Gly-Asp or RGD present on the FNIII repeat, binds to different integrin subunits and aids in the assembly of FN (Goodman, 2021). These non-covalent interactions help in forming a matured FN network.

Interaction of FN with cell surface receptors such as integrins and syndecans aid in cell adhesion and migration as well as determining cell shape and differentiation. Human mesenchymal stem cells (hMSCs) are known to utilize their own contractile forces and translate environmental cues such as variations in substrate rigidity into differential biochemical signals with the help of stretched FN fibers. hMSCs mediated osteogenesis was shown to be increased upon inhibition of αvβ3 integrin on relaxed FN fibers whereas osteogenesis was decreased upon inhibition of αvβ1 on stretched FN fibers. Thus, the mechanical strain of these FN fibers serves as a checkpoint, where they act as a crucial mechano-chemical signal converter that help the hMSCs to differentiate (Li et al., 2013). Osteogenic differentiation is known to be enhanced with increasing higher substrate stiffness ranging from 0.7 to 80 kPa. Interestingly, when polyacrylamide gels were coated with fibronectin, it stimulated osteogenic differentiation even at a lower substrate stiffness of 25 kPa, at a comparable differentiation rate to that of 80 kPa. These studies indicate that the mechanical properties of ECM components like fibronectin might play a significant role in determining the fate of stem cells (Rowlands et al., 2008). In embryonic stem cells (ES) fibronectin-mediated upregulation of the expression αvβ1 has been shown to promote the fate of cells towards a meso-endodermal lineage (Hayashi et al., 2007; Pimton et al., 2011). Additionally, in adult stem cells, fibronectin can promote differentiation towards a skeletal lineage as opposed to an adipogenic fate (Ogura et al., 2004; Martino et al., 2009; Wang et al., 2010). Interestingly, when differentiated 3T3 adipocytes were cultured on fibronectin, a reduction in the lipogenic gene expression was observed, suggesting that fibronectin also affects the fate of these cells (Spiegelman and Ginty, 1983). In mammalian skin, FN is also known to contribute to the wound healing process. The FN pericellular matrix assembled by fibroblasts senses strain caused by a disruption in tensile homeostasis during wounding. Mechanically regulated interaction between FN and collagen drives the deposition of collagen over FN matrices at the wound bed, which protects the FN matrix from the microenvironmental forces (Patten and Wang, 2021). Although it is known that the interaction between FN and collagen is mechanically regulated, the mechanism for the unfolding of the FN fibrils is less well understood (Gee et al., 2008; Ponmurugan and Vemparala, 2012; Früh et al., 2015; Szymanski et al., 2017a; Szymanski et al., 2017b).

#### 2.1.2 Matrix metalloproteinases

Matrix metalloproteinases (MMPs) are enzymes that cleave components of the ECM such as collagen and laminin, and play an important role in embryonic development, morphogenesis and tissue remodeling (Brenner et al., 1989; Matrisian and Hogan, 1990; Woessner, 1991; Tyagi et al., 1995). MMPs are a growing family of metallo endopeptidases and can be classified as collagenases, gelatinases, stromelysins, matrilysins and, enamelysins (Jabłońska-Trypuć et al., 2016). During development, MMPs help in regulating biophysical cues by degrading and modulating the stiffness of the ECM in different stem cell niches such as MSCs and HSCs (Caiazzo et al., 2016; Saw et al., 2019). MSCs play an active and significant role in bone regeneration due to their ability to migrate to injured sites and differentiate into osteocytes. Additionally, MMPs are also involved in bone regeneration as it has been shown that inhibition of MMPs, in particular MMP-13, led to perturbations in the migration, proliferation and osteogenic differentiation capabilities of the MSCs (Kasper et al., 2007). It was further demonstrated in the same study that mechanical loading also played a role in regulating the expression of MMPs, which may be an important factor in regulating levels of MMPs to direct MSC osteogenic differentiation. The conditional ablation of MMP-14 from mouse dermal fibroblasts results in enhanced accumulation of collagen I in the ECM, resulting in a fibrotic skin condition associated with an increase in stiffness and tensile strength. This study suggested that MMP-14 contributes to collagen remodeling in adult skin, and plays a crucial role in maintaining dermal homeostasis (Zigrino et al., 2016)**.** The migration of muscle stem cells (MuSCs) and progenitors to appropriate sites require physical interaction with its niche (Smith, et al., 2017). During this migration, cells experience mechanical resistance from the matrix, causing the cells to escape from their basement membrane. This process requires the expression of MMPs (MMP-1, MMP-14) from muscle progenitors to degrade the ECM in their path of migration (Wang et al., 2009; Xiaoping and Yong, 2009; Lund et al., 2014). Taken together, these studies show that MMPs play a critical role in modulating the physical properties of ECM to affect a variety of stem cell functions.

### 2.2 Cell‐substrate adhesions as niche factor

In multicellular organisms, adhesion molecules help to maintain tissue architecture, generate forces for cell movement and ensure the functionality of tissues by creating barriers (Vleminckx, 2017). Adhesion molecules bind to various ligands and receptors and the resulting signals are critical to many physiological processes such as cell growth, differentiation, contact inhibition and apoptosis (Vleminckx, 2017). The cytoplasmic domain of adhesion molecules bind indirectly to the cytoskeletal proteins such as actin and intermediate filaments, whereas their extracellular domain, via homophilic or heterophilic adhesion, interact with adhesion receptors on neighboring cells or ECM ligands. Furthermore, these adhesion molecules often cluster at specific restricted areas in the membrane, commonly termed as junctional complexes. The linkage to the cytoskeleton coupled with its clustering enhances the strength of adhesion forces nucleated by these adhesion molecules. Adhesion molecules are classified into five major groups based on their mode of interaction, function, structure and location. These groups are integrins, cadherins, selectins, proteoglycans and the immunoglobulin (Ig) superfamily (Samanta and Almo, 2015; Vleminckx, 2017). In this section, we will focus on the role of integrins, involved in cell-ECM interactions via the focal adhesions (FA) and cadherin, that mediate cell-cell interactions at the adherens junction (AJ) in stem cell functions. Junction complexes induced by cadherin and integrins can initiate signal transduction that facilitates niche signaling, in turn regulating stem cell properties such as self-renewal, proliferation and survival (Chen et al., 2013). The role of mechanical signals in regulating stem cell function, has been recently reviewed (Shih et al., 2011; Vining and Mooney, 2017; Naqvi and McNamara, 2020). However, the molecular mechanisms underpinning the mechanical activation of adhesion molecules and their roles in regulating stem cell fate are still less well understood.

#### 2.2.1 Integrin

Integrins are transmembrane heterodimeric receptors consisting of α and β subunits that function as a mechanical link between the cellular cytoskeleton and the ECM (Figure 1) (Sun et al., 2016). The engagement of integrins with their extracellular ligands initiates intracellular biochemical responses (Hynes, 1992; Arnaout et al., 2007). In mammals, there are eight β-subunits and 18 α-subunits resulting in 24 distinct integrin receptors (Hynes, 2002). These receptors bind to different ECM ligands such as laminin, fibronectin and collagen IV, with specific heterodimers playing important roles in stem cell maintenance in different tissue niches. For example, α_2_ integrin regulates the osteogenic differentiation in hMSCs and is upregulated in MSCs plated on stiffer matrices (42.1 kPa). On the other hand, the knockdown of α_2_ integrin in these MSCs decreased osteogenic induction, suggesting a role for this integrin in mechanotransduction during osteogenic differentiation (Shih et al., 2011). The reduction in quiescence of neural stem cells (NSCs) is associated with a lower expression of β1 integrin, which, in turn, results in a reduced interaction of the β1 integrin on the NSCs to the laminin-rich microenvironment (Shen et al., 2008; Nascimento et al., 2018). On the other hand, bifunctional hydrogels with different stiffnesses (2 and 20 kPa) that are engineered with laminin peptide (IKVAV) and poly-lysine led to neurogenesis in embryonic cortical progenitors and adult NSCs respectively via β1 integrin. These data suggest that substrate stiffness and ligand specificity of integrin contribute to different stem cell behavior (Farrukh et al., 2017). Although integrin is a well-known mechanotransmitter, integrin-mediated mechanosensing in regulating the stem cell microenvironment is still being explored.

#### 2.2.2 Integrin-associated proteins

Focal adhesions (FAs) are one of the key junctions involved in mechanosensing at the cell periphery. FAs comprise multimeric protein complexes which integrate both inside-out and outside-in signals and involve both mechanical and biochemical cues to regulate cellular functions (Vogel and Sheetz, 2006; Schiller and Fässler, 2013; Oakes and Gardel, 2014). FAs are nucleated by integrin receptors that form a mechanical link between the cytoskeleton and the ECM (Kanchanawong et al., 2010). Initially, integrins bind to the ligands found in the matrix, following which the cytoplasmic domain of the integrin subunit recruits multiple intracellular anchoring proteins such as talin, kindlin, paxillin, FAK, ILK, tensin etc (Figure 1). This anchorage can either result in the recruitment of vinculin, further linking this complex to the actin cytoskeleton, or result in direct engagement with the actin cytoskeleton (Alberts et al., 2002). Perturbations of these integrin-associated proteins have revealed their critical role in maintaining the stem cell niche.

Talin is an integrin-associated protein that is critical for integrin activation at the FA (Chinthalapudi et al., 2018). The lack of talin in embryonic stem cells in mice leads to the failure in the association of integrin with the cytoskeleton, resulting in embryonic lethality during gastrulation. This defect arises due to perturbed cell migration and cytoskeletal organization in the stem cell niche (Monkley et al., 2000). On the other hand, knockdown of talin in the testis results in disrupted adhesion within the germline stem cell (GSC) niche of *Drosophila.* This niche comprises stromal hub cells, GSCs and somatic cyst stem cells (CySCs). The adhesion between the hub cells and GSCs is required to maintain the undifferentiated state of GSCs, therefore the knock down of talin leads to an accumulation of differentiated GSCs (Raymond et al., 2009). A similar study in *C. elegans* showed that talin plays an important role in organizing the cytoskeleton, stabilizing adhesive contacts in muscle and modulating dynamic integrin signaling during migration (Cram et al., 2003).

Integrin-linked kinase (ILK) is a focal adhesion protein that interacts with the cytoplasmic tail of integrin β subunits and links them to the actin cytoskeleton. In the hair follicle stem cell (HFSC) niche, studies have pointed to the importance of ILK in maintaining quiescence of these cells. Deletion of ILK from the *epidermis* resulted in remodeling of the ECM and led to compensatory binding to laminin-511 over laminin-332 due to fragmentation and reduction of the latter. This imbalance caused an increase in Wnt and Tgf-β2 signaling and led to aberrant differentiation of HFSCs (Morgner et al., 2015). ILK has also been shown to be a key player during cardiomyogenesis. A decrease in ILK expression by siRNA resulted in a reduction in human fetal cardiomyogenesis, which could be attributed to an enhancement in Wnt signaling (Traister et al., 2012). Although the role of mechanotransduction in fetal cardiomyogenesis has not been directly investigated, it would be interesting to study if these dysregulations are mediated by forces generated by ILK. Lastly, mechanical stimulation of MSCs derived from mouse limb buds led to an increase in activation AKT/mTOR pathway which required ILK, ultimately resulting in an increase in differentiation and collagen expression in the niche of tendon cells (Mousavizadeh et al., 2020).

Focal Adhesion Kinase (FAK) is regulated by integrins and functions as a force sensor. Physically stretching cells causes cellular strain, increasing the force experienced at FAs, thereby activating FAK by phosphorylation at the Tyr397 residue (Schlaepfer et al., 1994; Calalb et al., 1995; Polte and Hanks, 1997). Forces experienced in focal adhesions are positively correlated with Tyr397 phosphorylated in FAK, suggesting that lower force results in reduced FAK activation (Torsoni et al., 2003). FAK activity is positively regulated by Rho-associated kinases and non-muscle myosin II. Cytoskeletal contractility along with FA formation decreased upon inhibition of the Rho-associated kinases (Seo et al., 2011). FAK activation is required for osteogenic differentiation (Salasznyk et al., 2007; Shih et al., 2011; Mathieu and Loboa, 2012) and is further correlated with myosin II being involved in osteogenic differentiation. Additionally, force-mediated activation of FAK results in activation of Rho which can then result in force-mediated adult stem cell differentiation (Thompson et al., 2012).

Tensin is known to interact with actin as well as integrins at the FAs and establishes a crucial connection between the cytoskeletal network and the ECM. Tensin three is widely expressed in tonsil-derived MSCs (TMSCs), where it plays an important role in the proliferation and differentiation of TMSCs by regulating the levels of β1-integrin (Park et al., 2019). The Ivaska’s group demonstrated the role of the energy sensor AMP-activated protein kinase (AMPK) that worked in association with tensin to increase the activity of integrins. Mechanistically, AMPK negatively regulates tensin 1 and tensin 3, which in the absence of AMPK, binds to β1-integrin, thereby regulating integrin dependent processes like cell adhesion, mechanotransduction and cell matrix formation (Georgiadou et al., 2017). Although not much is known about the role of tensin in mechanotransduction, future studies will be needed to address its role in stem cell homeostasis.

Kindlin is a scaffold protein, required for several protein-protein interactions in the FA complex. Recent studies have identified Kindlin 2 as a critical mechanosensor in MSCs. Kindlin 2 plays an important role in regulating YAP/TAZ expression and localization to regulate the fate of the MSCs. Mechanistically, kindlin-2 interacts with Myosin Light Chain Kinases (MLCKs), which controls the MSC microenvironment and intracellular signaling through RhoA activation and phosphorylation of MLC. This interaction controls the actin organization and YAP/TAZ phosphorylation, ultimately controlling YAP/TAZ mediated gene expression to regulate the MSC differentiation (Guo et al., 2018). Kindlin-2 is also reported to regulate chondrogenesis, osteogenesis and osteocyte survival. Deleting kindlin-2 from osteocytes, which are the mechanosensors of bone, has been shown to impair bone homeostasis, disrupt FA formation, cytoskeletal organization and cell orientation in bone. Loss of kindlin 2 also induced apoptosis in osteocytes and resulted in the abnormal expression of sclerostin, a negative regulator of bone formation under mechanical stimulation (Qin et al., 2021).

Paxillin is a phosphotyrosine-containing docking protein that is primarily localized to the FA complex (Glenney and Zokas, 1989; Schaller, 2001). Paxillin interacts with FAK and other signaling molecules, associated with integrin signaling and membrane trafficking (Wade et al., 2002). The mechanosensing properties of paxillin are enabled by its binding to activated vinculin that stabilizes the FA-cytoskeleton interaction. Both paxillin null mouse embryonic stem (ES) cells and paxillin null differentiated cells display defects in focal adhesion and cell spreading, coupled with an observed reduction in FAK phosphorylation (Wade et al., 2002). As studies have only begun to scratch the surface, research demonstrating a direct link between mechanotransduction and maintenance of the stem cell niche via paxillin requires further investigation.

The FA protein vinculin, which is associated with the actin cytoskeleton, is also present at the cell-cell junction (AJ). Vinculin is a well-known mechanotransducer that directly interacts with talin, α-actinin and paxillin at the FAs and α-catenin at the AJs. Various studies have shown the mechanotransduction role of vinculin at the cellular level. Only a few studies have highlighted the mechanotransduction role of vinculin in maintaining the SC niche. A study by Holle et al. showed that KD of vinculin in hMSCs resulted in a reduction of cytoplasmic and FA vinculin (Holle et al., 2013). Vinculin KD also led to a reduction in myoblast determination protein 1 (MyoD) expression in the nucleus. MyoD is the transcription factor which induces stem cell differentiation into a myoblast lineage. Re-expression of the full length or head domain of vinculin was sufficient to restore MyoD expression allowing cells to return to their myogenic state. Additionally, vinculin KD cells displayed hampered durotaxis or cell migration towards a stiffer, more myogenic-permissive matrix as opposed to control cells, which assembled preferentially at the myogenic-permissive matrix. Another transcription factor, Myogenic factor 5 (Myf5), regulates muscle differentiation along with MyoD. Studies have shown that vinculin KD resulted in the loss of stiffness-mediated expression of MyoD and Myf5 hindering myogenic differentiation. Recently, our group has shown the role of vinculin in maintaining HFSC quiescence (Biswas et al., 2021). Usually HFSCs are maintained in a quiescence state which is achieved by the cell to cell contact mediated inhibition of proliferation (Fan and Meyer, 2021). Conditional KO of vinculin in murine *epidermis* resulted in mechanically weak AJs which failed to sequester YAP at the junctions and led to increased proliferation of the HFSCs. Mechanistically, our work showed that, in contact-inhibited (quiescent) bulge stem cells, vinculin was critical in maintaining α-catenin in a stretched/open conformation reinforcing the junction which in turn kept YAP sequestered at the AJs. These studies highlight the crucial role of mechanotransduction through vinculin at the FAs and AJs for maintaining the SCs niche.

### 2.3 Cell-cell adhesions as niche factor

So far, we have discussed how integrins and integrin associated proteins at the FAs play an active role in mechanotransduction. Cells also interact with each other by forming cell-cell junctions including AJs, desmosomes, tight junctions and gap junctions (Figure 1). Among these, AJs are the key adhesion structures that interact with the cytoskeleton of neighboring cells to enable mechanical force transduction (Pannekoek et al., 2019).

#### 2.3.1 Adherens junction

AJs mediate adhesion through homotypic interactions of cadherins such as E-cadherin which are calcium-dependent, transmembrane proteins (Figure 1). E-cadherin associates with the catenin family of proteins such as β-catenin, α-catenin, and p120 via its cytoplasmic domain. This forms an E-cadherin-catenin complex that interacts with the F-actin cytoskeleton by either direct binding to α-catenin or an indirect association via vinculin (Koirala et al., 2021). AJs regulate cell-cell adhesion within the stem cell niche thereby affecting stem cell fate.

##### 2.3.1.1 E-cadherin

E-cadherins are critical to the formation and maintenance of cell junctions, and for the proliferation, survival and differentiation of cells to maintain tissue integrity. Cell-cell adhesion mediated by E-cadherin is linked to signaling pathways that transduce signals to the cytoplasm and nucleus (Stepniak et al., 2009). Studies have also shed light on the importance of E-cadherin mediated stem cell niche maintenance in regulating the self-renewal of NSCs (Karpowicz et al., 2009). E-cadherin has also been shown to be crucial in maintaining the pluripotency of mESCs as the disruption in E-cadherin leads to increased Epithelial-Mesenchymal Transition (EMT) and differentiation of these cells (Redmer et al., 2011). The deletion of the E-cadherin gene in mESCs leads to the failure of cell compaction in the embryonic blastocyst that is important for maintaining the epithelial subcellular structures i.e., cellular junctions, during early mammalian development (Larue et al., 1994). Additionally, E-cadherin induces conformational changes in α-catenin through the transmission of adhesion forces from the cell-cell junctions. This leads to strengthened binding of α-catenin to F-actin and the recruitment of vinculin to the site where these forces are applied.

##### 2.3.1.2 β-catenin

In epithelial tissue, β-catenin is one of the crucial components of the AJ, where it links cadherins to the cytoskeleton of the cell, thus establishing cell adhesion. Subcellular localization of β-catenin is known to determine its function within the cell (Dietrich et al., 2002). When β-catenin localizes in the cytoplasm or nucleus, it functions as a major signaling hub for the Wnt/β-catenin signaling pathway, where it acts as a transcriptional co-activator of T cell factor/lymphoid enhancer factor family (TCF/LEF) target genes (Kim et al., 2019). β-Catenin also acts as a mechanotransducer (Benham-Pyle et al., 2016), where mechanical cues induce its nuclear accumulation and enhance transcriptional activity (Benham-Pyle et al., 2015). Additionally, it has also been reported that mechanical perturbation of *Drosophila* embryos causes increased β-catenin signaling and upregulation of its downstream target genes (Farge, 2003). The Wnt/β-catenin signaling pathway is known to respond to ECM stiffness (Du et al., 2016), which ultimately affects cellular functions such as proliferation, differentiation, migration, genetic stability, apoptosis, and stem cell renewal (Pai et al., 2017).

Jansen and co-workers demonstrated that the biphasic effects of mechanical loading on β-catenin resulted in negative effects on Wnt signaling in osteoblast differentiation and mineralization (Jansen et al., 2010). Murine embryonic stem cells (mESCs) showed enhanced expression of various pluripotency markers Oct4, Sox2, Nanog, LIF (also known as mechanopluripotency) when cultured in a stirred suspension bioreactor. Mechanical cues promoted by fluid shear induces the nuclear translocation of β-catenin. This causes a concomitant increase of c-Myc, which is an upstream regulator of these pluripotency markers, ultimately promoting mESCs mechanopluripotency (Nath et al., 2021). This study indicates that mechanotransduction through the AJ complex is critical for mESC pluripotency maintenance. In inner ear progenitor cells (IEPCs), external mechanical signals from ECM can be transduced through the RhoA-YAP-β-catenin signaling cascade to regulate its survival, proliferation and differentiation (Xia et al., 2020). Taken together, β-catenin functions as a mechanical regulator that controls cellular functions important for maintaining stem cell homeostasis.

##### 2.3.1.3 α-catenin

α-Catenin is another key molecule in the AJ, that links to the actin cytoskeleton and the cadherin complex via β-catenin (Niessen & Gottardi, 2008). The association of α-catenin with the actin cytoskeleton, allows it to sense the changes in tissue tension during cytoskeletal contraction. α-Catenin thus acts as a mechanosensor, where it converts mechanical cues into biochemical signals that control cell proliferation and cell death (Yonemura et al., 2010; Sarpal et al., 2019). The Conditional KO of α-catenin in the skin *epidermis* results in abnormal hair morphogenesis, along with defective AJ formation and cell polarity (Vasioukhin et al., 2001). Additionally, the loss of α-catenin from the *epidermis* results in hyperproliferation of the interfollicular *epidermis* (IFE) and the presence of multinucleated cells. This, in turn, leads to a precancerous skin condition with characteristics similar to squamous cell carcinoma. A follow-up study by Vasioukhin’s group in 2011 showed that specific deletion of α-catenin from the HFSC compartment led to the development of squamous cell carcinomas. This was due to the direct interaction of the transcriptional co-activator YAP1 with α-catenin. The cKO of α-catenin from the HFSC leads to translocation of YAP to the nucleus, thereby promoting cell proliferation and forming pre-cancerous lesions (Silvis et al., 2011). It was later demonstrated that the interaction of 14-3-3 and the PP2A phosphatase with α-catenin led to the phosphorylation of YAP1, sequestering it to the junctions, and preventing hyperproliferation (Schlegelmilch et al., 2011). This suggested that α-catenin acts not only as a tumor-suppressor, but also as a sensor of cell density in the skin. Knockdown of α-catenin in human embryonic SCs (hESCs) results in the induction of endodermal differentiation due to ubiquitylation and proteolysis of β-catenin. This inhibits repression of Wnt target genes in the transformed cells (Choi et al., 2013). Association of α-catenin with the adenomatous polyposis coli (APC) tumor suppressor, a component of the Wnt/β-catenin pathway, was shown to control the ubiquitination of β-catenin via the APC destruction complex. Therefore, the reduction of α-catenin levels in hESCs leads to a failure in proteolytic destruction of β-catenin, inducing differentiation of the endoderm. Studies on the process of mechano-sensing of α-catenin have only recently started to be investigated. However, the force-dependent conformational change of α-catenin has been shown to activate proteins that facilitate binding with the dynamic actin cytoskeletal network.

##### 2.3.1.4 p120

p120 catenin is one of the catenins that interacts with E-cadherin at the AJ (Reynolds et al., 1994; Kourtidis et al., 2013). The localization of p120 catenin from the AJ to the cytoplasm upon mechanical stress sensing results in the increased turnover of E-cadherin, which in turn promotes wing development in *Drosophila*, suggesting the importance of p120 as a mechanotransducer at the junctions (Iyer et al., 2019). Several other studies have also described the role of p120 in SC maintenance. During early embryogenesis in mice, p120 null mutant mouse embryonic SCs (mESCs) failed to differentiate completely. The absence of p120 in mESCs leads to the destabilization of E-cadherin at the AJs, causing defective formation of the primitive endoderm (Pieters et al., 2016). A study on mESCs by Pierre D. McCrea’s group showed that p120 catenin negatively regulates a repressive transcriptional complex, RE1-silencing transcription factor/co-repressor (REST/coREST), that plays a critical role in stem cell fate determination. Direct binding of p120 to the REST/coREST complex prevented proper differentiation of the mESCs towards neural fate, thereby uncovering a role for p120 in modulating SC differentiation (Lee et al., 2014). p120 catenin acts as a mediator in cellular positioning and organ patterning during fate determination of pancreatic progenitors in mice by regulating the differential expression of E-cadherin. This in turn drives cellular motility and directs cells towards specific niches to determine their cellular fate (Nyeng et al., 2019). Taken together, p120 not only acts as a mediator for E-cadherin function but also plays an important role in lineage commitment. Further studies are required to fully understand how mechanotransduction by p120 regulates the SC niche.

#### 2.3.2 Desmosomes

Desmosomes are intercellular cadherin-mediated cell-cell junctions that couples to the intermediate filaments and confer mechanical stability when cells are exposed to tensile and mechanical stress (Figure 1). Specifically, desmosomal cadherins are formed by desmogleins 1-4 and desmocollin 1–3, which bind to armadillo proteins plakoglobin and plakophilins via their cytoplasmic tails and subsequently binds to intermediate filaments via Desmoplakin (Angulo-Urarte et al., 2020). The KO of DSG2 results in peri-implantation lethality and also led to a decrease in embryonic stem cell proliferation (Eshkind et al., 2002). DSG2 was shown to be important for regulating the self-renewal and differentiation capacity of pluripotent stem cells (PSCs). Depletion of DSG2 using monoclonal antibodies in hPSCs resulted in decreased proliferation and reduced expression of pluripotency markers (Park et al., 2018). These results implicate a role of desmosomes in regulating stem cell proliferation and differentiation, however, more work is needed to ascertain how mechanical signals regulate this process. Different compositions of desmosomes lead to a difference in the strength of cell-cell adhesion in different layers of the skin, which in turn, correlates with different levels of proliferation and differentiation. The basal proliferating keratinocytes express desmocollin 2/3 (DSC2/3) and desmoglobin 2/3 (DSG2/3) which displayed the weakest binding out of all the different desmosomal proteins. In contrast, the differentiated cells in the suprabasal layers such as the cornified layer contain a higher composition of DSC1 and DSG1/4, which provided the strongest binding strength. This correlates with the need for differentiated keratinocytes to form strong and stable cell-cell adhesions to maintain a physical barrier that protects the *epidermis* from mechanical and chemical stresses (Harrison et al., 2016; Green et al., 2019). Taken together, studies on desmosomes have begun to shed some light in understanding its role along with other adhesion proteins in maintaining the proliferative capacity of stem cells in its niche. However, more studies are needed to fully understand its role in mechanical transduction and maintaining other stem cell niches.

#### 2.3.3 Tight junctions

Tight junction (TJ) proteins are a branching network of intercellular adhesion complexes that controls the permeability of the tissue. TJs are present on epithelial and endothelial cells (Figure 1). In the epithelium, tight junction structures form partitions between the apical and the basolateral domains that prevent the intermixing of both the transmembrane components, thereby supporting cell polarity. TJ proteins are categorized into two groups depending on their functionality: integral transmembrane protein and peripheral membrane proteins or plaque proteins. Four transmembrane proteins namely claudins, occludin, tricellulin and junctional adhesion molecule (JAM) aids in forming tightly regulated networks between the peripheral membrane proteins. On the other hand, peripheral adapter proteins such as zona-occludens 1 (ZO-1), ZO-2 and ZO-3 play a role in connecting the transmembrane proteins to the cytoskeleton and other signaling molecules (Lee et al., 2018). Differences in expression of these TJ proteins play an important role in regulating the barrier integrity of the membrane.

Studies have shown that TJ proteins play an important role in controlling the biological functions of several stem cells. For example, occluding junctions have been shown to play a novel role in regulating the niche of the hematopoietic stem cells when induced with a bacterial infection (Khadilkar et al., 2017). In *Drosophila*, an immune response is triggered upon infection that leads to barrier breaks due to modulations in the occluding junctions. This in turn helps in prohemocyte differentiation that eventually induced immune cell production, thereby activating the immune response. TJ proteins like occludins, ZO-1, caludins present in human NSCs (hNSCs) are known to help in the formation of hNSC clusters known as neurospheres. The formation of these neurospheres are critical for maintaining the stemness of hNSCs. TJ proteins were downregulated upon induction of differentiation in NSCs, further corroborating the fact that they are important in maintaining the stemness of the NSCs (Watters et al., 2015). This suggests a plausible connection between TJ protein levels and their regulation of the NSC niche. However, further studies are required to establish the direct interaction of NSCs and the TJ protein complex that might play a role in this cluster formation.

## 3 Implication of adhesion molecules in diseases

Several diseases have been linked to changes in the expression or function of various adhesion molecules (Table 1).

**TABLE 1:** Diseases associated with cell adhesion molecules.

Type of cell adhesion molecule	Disease	Mechanism of the disease	Distribution	Therapy
Focal adhesion proteins (cell-substratum)	Junctional Epidermolysis Bullosa	Laminin 5, α3β1 integrin and α6β4 integrin	Basement membrane of skin	*ex vivo* cell and gene therapy De Rosa et al. (2014), Hirsch et al. (2017), Mavilio et al. (2006)
	Dystrophic Epidermolysis Bullosa	Collagen VII	Below the basement membrane of skin	Hematopoietic cell transplantation (HCT)
				Petrof et al. (2015), Rashidghamat and McGrath (2017), Tolar and Wagner (2013)
				(https://clinicaltrials.gov/ct2/show/NCT02582775) (https://clinicaltrials.gov/ct2/show/NCT01033552)
	Mammary Gland Cancer	Integrin β1, Collagen V, FAK	Mammary Gland	Targeting signaling pathways in cancer stem cells (CSCs)
				Yang et al. (2017)
				Targeting integrin β1 in mice xenograft models Park et al. (2006), White et al. (2004)
	Atherosclerosis	Collagen I, III	Heart	MSC therapy: Gorabi et al. (2019), Lin et al. (2020), Zhang et al. (2014)
	Osteoporosis	Collagen I	Bone and skeletal	MSC therapy Arjmand et al. (2020), Jiang et al. (2021), Kangari et al. (2020), Pouikli et al. (2021)
	Alport Syndrome	Collagen IV	Kidneys (Glomeruli)	Stem cell Therapy: LeBleu et al. (2009)
				Stem Cell Therapy - Alport Syndrome News
	Ullrich congenital muscular dystrophy	Collagen 6α1, α2, α3	Skeletal muscles	Collagen supplementation: Takenaka-Ninagawa et al. (2021); Yonekawa and Nishino (2015)
	Glanzmann thrombasthenia	Integrin α11b3	Platelets	Hematopoietic stem cell therapy
				Crone and James (2019), Connor et al. (2008)
				https://rarediseases.org/rare-diseases/glanzmann-thrombasthenia/
	Leukocyte Adhesion Deficiency 1	Integrin β2	Leukocytes (T and B cells)	Hematopoietic stem cell therapy
				Qasim et al. (2009)
	Leukocyte Adhesion Deficiency 3	Kindlin 3	Leukocytes (T and B cells)	Hematopoietic stem cell therapy
				Stepensky et al. (2015)
	Fibronectin glomerulopathy	Fibronectin	Kidney	Symptoms are treated currently Ishimoto et al. (2013)
	Williams Beuren syndrome	Elastin	Lungs, blood vessels, skin, gastrointestinal, genitourinary	Symptoms are currently treated Duque Lasio & Kozel (2018)
	Laminin-α2-related congenital muscular dystrophy (LAMA2-CMD)	Laminin α2	Muscle	Protein replacement therapy Barraza-Flores et al. (2020)
	Desmosomes (cell-cell junction)	Epidermolysis Bullosa Simplex	Desmoplakin	Intra-epidermal region	Gene therapy Marinkovich and Tang (2019)
			Arrhythmogenic cardiomyopathy	Plakophilin-2, Desmoplakin, Desmoglein-2 and Desmocollin-2	Heart	Stem cell therapy Liu et al. (2021) , campos de carvalho et al. 2021
	Adherens Junction (cell-cell junction)	Oral cancer, Basal cell adenoma (BCA), Mucoepidermoid Carcinoma (MEC), Adenoid Cystic Carcinoma, Colorectal cancer	β-catenin	Oral cancer: Mouth BCA: Skin	Stem cell therapy Suma et al. (2015), Baniebrahimi et al. (2020), Shahoumi (2021), Yuan et al. (2021)
				MEC: Salivary gland	
				Adenoid Cystic Carcinoma: Salivary gland, Head and neck	
				Colorectal cancer: Colon	
		Squamous cell carcinoma	α-catenin	Head and Neck, Skin	Stem cell Therapy Affolter et al. (2021), Chen and Wang (2019)
		Cardiomyopathy	αE-catenin	Heart muscles	Stem cell therapy: Diaz-Navarro et al. (2021), Jiao et al. (2014), Tripathi et al. (2021)
		Hereditary Diffuse Gastric Cancer (HDGC), Breast cancer, Epithelial ovarian cancer	E-cadherin (CDH1)	HDGC: Stomach	HDGC: Currently, no SC therapies are available
				Breast cancer: Breast	Breast cancer: Stem cell Therapy: Khan et al. (2021), Kitamura et al., 2021
				Epithelial ovarian cancer: Ovaries	Epithelial ovarian cancer: Currently, no SC therapies are available
		Dilated cardiomyopathy (DCM) and hypertrophic cardiomyopathy (HCM)	Vinculin	Heart	DCM: Stem cell-based therapies Catelain et al. (2013), Diaz-Navarro et al. (2021)
				Stem cell breakthrough unlocks mysteries associated with inherited heart condition -- ScienceDaily Regenerative Therapy for Cardiomyopathies ‐PMC (nih.gov)
				Stem cell therapy Wang et al. (2018)
				Gene therapy: Vinculin Over expression Kaushik et al. (2015)
		Atopic dermatitis	p120 catenin	Cutaneous disorder, skin	Mesenchymal stem cell Therapy: Daltro et al. (2020), Guan et al. (2022)
					www.sciencedaily.com/releases/2016/06/160607080935.htm
Tight Junctions (cell-cell junctions)		Inflammatory bowel disease (IBD)	Claudins 2, 3,4 5,7,8	Intestine	Stem cell therapy Sawada (2013); Shimizu et al. (2019)
		Congenital deafness	Claudin 14 and Claudin 11	Ears	IPSC based therapy: He et al. (2021), Peng et al. (2014)
			Multiple Sclerosis	Claudin 11	Central nervous system	Currently, no SC therapies are available

### 3.1 Inflammatory bowel disease

One of the roles of the intestinal epithelium is to prevent the exposure of the gastrointestinal tract to foreign antigens and harmful microbiota. This is achieved by the formation of tightly regulated cell junctions and organization of the epithelium to establish a barrier. This epithelial barrier integrity is maintained by the TJ proteins that consist of both transmembrane and peripheral membrane proteins like occludin, zonula occludens and claudins that are linked to the cytoskeleton of the cell (Landy et al., 2016; Chelakkot et al., 2018). Defects in maintaining this barrier function leads to pathological conditions like inflammatory bowel disease (IBD).

IBD is a chronic inflammatory disease that causes severe inflammation of the gastrointestinal tract. The disease is mainly categorized into two types depending on the part of the inflamed intestine: Ulcerative colitis (UC) and Crohn’s disease (CD) (Chelakkot et al., 2018; Binienda et al., 2020). The characteristic symptoms of IBD are a leaky gut that is caused by the apoptotic and ulcerative intestinal epithelium, resulting in severe inflammation. Previous studies have shown that over-expression of a TJ protein claudin 2 in UC patients increased pore formation in their epithelium. On the other hand, claudins 3, 4 and 7 which help in tightening the epithelial junctions were found to be reduced in these patients (Prasad et al., 2005; Oshima et al., 2008). This suggested differential expression of several TJ proteins might be a major cause of increased permeability of the intestinal epithelium that leads to defective barrier function. Similarly, in CD, perturbed organization of the TJ proteins was observed in inflamed areas. Several studies showed claudin 2 to also be upregulated in the CD patients with a concomitant downregulation of claudin 3,5,8 (Kucharzik et al., 2001; Zeissig et al., 2007; Das et al., 2012). In addition to TJ proteins, AJ proteins have also been shown to be involved in a relapse of this disease. Downregulation of the E-cadherin-catenin complex led to the loss of cell-cell junctions in the gut that exposed the luminal content to the immune cells thereby increasing the relapse of CD (Wyatt et al., 1993; Arnott et al., 2000).

Treatment options for IBD have substantially improved over the past few decades. Several drugs have been developed to target interleukins (ILs), tumor necrosis factor (TNF) as well as JAK signaling that regulates several cytokines, for treating both the types of IBD. Even though there has been advancement in the drug discovery and delivery process, some patients still do not respond to the drugs and display a relapse of the symptoms. SC based therapy is a promising treatment option that has started to develop. Somatic SCs such as HSCs and MSCs have already been used for treating IBD patients (Shimizu et al., 2019). The HSC transplantation process requires rebooting the immune system by lymph ablation followed by reconstruction of the immune system. A pilot study based on autologous HSC transplantation showed regaining of responsiveness to the drugs as well as sustained remission in about 80% of the patients suffering from refractory CD (López-García et al., 2017). Even though there was an improvement after the transplantation, CD relapsed in most of the patients in a span of 5 years. In addition, severe adverse events occurred in many patients who received HSCT compared to the control group. Taken together, this study suggested HSCT to be one of the methods that can be used for treating patients with severe CD but requires improvement compared to the conventional methods.

Mesenchymal stem cell therapy is another technique that has been used for treating active CD fistula (Misselwitz et al., 2020). MSCs have been reported to show immunosuppressive behavior as well as decreased the effect of the lymphocytes, thereby modulating the immune system (Aggarwal and Pittenger, 2005; Corcione et al., 2006; Ren et al., 2008). Clinical studies have already been conducted using bone marrow derived MSC transplantation (MSCT) as well as adipose derived MSC transplantation (ASCT) (Shimizu et al., 2019). In a phase 2 trial of allogeneic mesenchymal stromal cell transplantation, the Crohn’s disease activity index (CDAI) was reduced in patients suffering from luminal CD (Forbes et al., 2014). Almost 53% of the patients had clinical remission within 42 days of receiving the treatment. In this study, only one case of severe adverse effect was reported, which led to the occurrence of stage 1 adenocarcinoma after 3 weeks of the treatment. Even though MSCT seemed better than the HSCT, long term efficacy of this treatment to maintain long-term disease remission needs further investigation. On the other hand, in a phase 3 randomized, double-blind study, ASCT was shown to be an effective treatment for patients with complex perianal fistula associated with Crohn’s disease (Panés et al., 2016). Among the 212 patients randomly divided into treatment and placebo groups, almost 50% of the ASCT treated patients attained remission. Adverse effect cases that included anal abscess and proctalgia were almost similar in number for both the treated and placebo patients. This suggested both ASCT and MSCT to be safer treatment options for IBD patients who failed to respond to conventional treatments.

In 2012, Yui et al. developed intestinal organoid models and transplanted intestinal stem cells (ISC) in mice with colitis (Yui et al., 2012). Based on this study, Ryuichi Okamoto’s group is now using ISC transplantation (ISCT) to study this potential treatment method to cure patients with refractory IBD. ISCs have been isolated from patient derived biopsies that are now being tested for therapeutic potential (Shimizu et al., 2019). These cells have the potential to proliferate and reestablish the wound bed in these patients, thereby protecting the organ from mechanical and pathogenic insults. This method is awaiting clinical trials but has the potential to directly cause mucosal healing. In addition to the stem cell-based treatments, given the importance of maintaining barrier function in IBD, identifying the mechanism of the cell-cell junctions regulation might aid in improving these therapeutics.

### 3.2 Epidermolysis bullosa

Epidermolysis bullosa (EB) is a group of rare diseases caused by mutations in genes that encode for both ECM and focal adhesion proteins and is estimated to affect 1 in 30,000 people worldwide (Bruckner et al., 2020). It is characterized by fragile skin and severe blistering and lesions that can occur from as early as childbirth. The most common type of EB is Epidermolysis Bullosa Simplex (EBS), and can be caused by autosomal recessive mutation of the desmoplakin gene in the suprabasal cells or autosomal dominant mutations in the keratin 5/14 genes in the basal cells. Junctional Epidermolysis Bullosa (JEB), which occurs due to an autosomal recessive mutation in an array of ECM and hemidesmosome proteins such as Laminin-332 and α6β4 and BP230 integrin along the basement membrane. Dystrophic Epidermolysis Bullosa (DEB) occurs due to a mutation in Collagen VII, which is localized below the basement membrane (Bruckner-Tuderman, 2019). These molecular mutations impair the structure and functional integrity within highly specialized interfaces of the skin, that are important for cell adhesion, tissue repair and barrier function. Ultimately, this leads to a diminished resistance to mechanical stress and shearing forces, which causes subsequent cell and tissue damage (Uitto and Richard, 2004; Fine et al., 2008; Bruckner-Tuderman et al., 2013).

EB poses as a potentially life-threatening disease as the perpetual and progressive scarring triggered by skin blistering and lesions leads to chronic wounds. These chronic wounds are coupled with increased bacterial colonization, fibrosis, inflammation, and a systemic development of cutaneous squamous cell carcinoma (Guerra et al., 2017). Mutations in the genes are not restricted to the skin but also in other epithelialized (gastrointestinal, urogenital tract, respiratory) or mesenchymal (skeletal muscle) organs (Prodinger et al., 2019). EB with Pyloric atresia in patients affects both the skin and digestive tract, causing severe blistering and also an obstruction of the pylorus (Nakano et al., 2001). There are no direct cures for any of the EB subtypes, and current treatment strategies are only useful in managing the wounds and pain caused by EB. Numerous preclinical research and developments using gene correction, protein replacement and cell-based treatments such as bone marrow transplantation and mesenchymal stem cell therapy have pointed to new therapeutic avenues and have entered early clinical trials.

Stem cells present a promising avenue to reverse some of the damage caused by Recessive Dystrophic Epidermolysis Bullosa (RDEB) and early research in animal models suggest that hematopoietic cell transplantation (HCT) from WT donor-derived cells to RDEB mice improved its survival and skin strength (Tolar et al., 2009). These positive results spurred the development of a small clinical trial involving allogeneic HCT in 6 children with RDEB. Overall, the trial showed promising results as 5 out of 6 of the patients displayed an increase in collagen VII deposition in the Dermal Epidermal Junction (DEJ), improved wounding healing and a decrease in blister formation (Tolar and Wagner, 2013). Additional trials have been conducted with varying outcomes (Petrof et al., 2015; Rashidghamat and McGrath, 2017). Current phase 2 clinical trials include further testing of healthy donor mesenchymal stem cell infusions into EB patients. These developments are promising and provide potentially better therapeutic treatments forEB. (https://clinicaltrials.gov/ct2/show/NCT02582775; https://clinicaltrials.gov/ct2/show/NCT01033552). Additionally, studies from other groups have successfully employed *ex vivo* cell and gene therapy to treat intermediate JEB caused by mutations in Laminin β3. In an initial study, epidermal stem cells from an adult patient affected by Laminin β3 deficient JEB were transduced with a retroviral vector expressing Laminin β3 cDNA encoding functional Laminin β3 and applied to the affected skin area as cohesive epidermal sheets made from a plastic substrate (Mavilio et al., 2006). The study proved to be efficacious during a 6.5 years follow up, where the *epidermis* became fully functional and restored with no adverse reaction (De Rosa et al., 2014). This positive response spurred further testing on a 7-year-old boy in 2015 with EB and epidermal loss in 80% of his body using the same gene therapy to produce functional laminin 332 from transgenic cultured *epidermis* using a fibrin substrate (Hirsch et al., 2017). Similarly, the patient displayed good results after the treatment, with his newly formed *epidermis* expressing normal levels of laminin 332, displaying a functional basement membrane that remained resistant to mechanical stress and blistering. In the long run, gene therapy to correct mutations causing EB might prove useful. Further scrutiny and hopes can be pinned onto these 2 patients as they continue to undergo observational studies to monitor the efficacy of this treatment method (https://clinicaltrials.gov/ct2/show/NCT05111600).

### 3.3 Breast cancer

Breast cancer is the most common cancer occurring in women globally. Annually, about 1.7 million new cases are recorded in the world (Hai-Ying et al., 2020). The mammary gland is the milk-producing organ in animals, which continuously develops after birth and fully differentiates upon pregnancy and lactation. During postnatal development and onset of reproductive cycles, the mammary gland is dynamically remodeled and undergoes morphological changes due to changes in cell proliferation, differentiation, migration and apoptosis. This inherent plasticity has been suggested to increase the susceptibility of the organ to carcinogenesis (Paavolainen and Peuhu, 2021).

Mechanical signals that cells receive from their surroundings are emerging as key players and contributors to tumor progression (Humphrey et al., 2014; Panciera et al., 2017). These mechanical signals change with the composition of the ECM during mammary development. Tumor progression of mammary cells can be characterized by increased ECM deposition, also known as desmoplasia. Collagen V is increased in desmoplastic stroma in human breast carcinoma (Barsky et al., 1982). Clinically, desmoplastic stroma is associated with a 4–6 fold increased relative risk of developing breast carcinoma, with poor prognosis (Boyd et al., 2001; Guo et al., 2001)**.**

Recent studies have begun to highlight the importance of external mechanical signals that mammary gland cells receive in driving tumorigenesis. Initially, it was shown that primary luminal mammary gland cells were able to form self-renewing colonies in the presence of oncogenic factors such as EGFR and activated HER2 (Panciera et al., 2020). This led to the development of solid organoids composed entirely of K8+ luminal cells which are a hallmark of human HER2+ breast cancer. However, when these cells were plated on a soft adhesive hydrogel of 0.5 kPa which phenocopies the external environment of the normal mammary gland, the presence of the same oncogenic factors were insufficient to develop into self-renewing solid organoids reminiscent in breast cancers. However, when the primary luminal cells were plated on the hydrogels of higher rigidity (40 kPa), the growth of these organoids was observed. Mechanistically, this was shown to be due to the mechanical cues conferred from a stiffer environment, that ultimately led to increased YAP/TAZ signaling and increased proliferation and self-renewal (Panciera et al., 2020). YAP activation also causes remodeling of the surrounding ECM and this favored tumor spreading (Calvo et al., 2013).

Other studies have also begun to highlight the role of various cell adhesion molecules in the development of breast cancer, such as the activation of intracellular FAK (dos Santos et al., 2012; Provenzano and Keely, 2009). In particular, change to a stiffer ECM has been found to correlate with increased FAK activation and increase tumorigenesis in mammary cells. Integrin αvβ3 was also found to be significantly elevated in metastatic tumors compared to primary pancreatic and breast tumors. Mechanistically, this seemed to occur by the recruitment of Src kinase that promotes migration of the cancer cells (Ghajar & Bissell, 2008; Desgrosellier et al., 2010). Studies from multiple groups have described potential therapeutic modalities such as integrin β1 blocking antibodies, since tumors derived from human xenograft models and transgenic models displayed significant growth inhibition upon treatment with an integrin β1 inhibitory antibody (White et al., 2004; Park et al., 2006). As the ECM is increasingly recognized as the master regulator of cell response and behavior, an increase in the knowledge and understanding of the dynamic role that ECM plays in cancer biology, both in terms of its architectural complexity and how this affects the tumor progression in breast cancer, will be valuable. Biomimetic models such as the decellularized ECM recapitulates the complex ECM microenvironment of the mammary glands and can be useful tools to allow a better understanding of cell-ECM interactions. Although these approaches to treating breast cancer are still new, it may shed light on the tumorigenesis of the mammary gland cancer and open new treatment paradigms (Wishart et al., 2020; Tamayo-Angorrilla et al., 2021).

## 4 Discussion, perspectives and future directions

The connection between cells and ECM through FA and the cell to cell junction through AJ, both serve as mechanosensitive hubs. In this review, we have discussed how mechanotransduction through these hubs contribute to maintaining various stem cell niches. Additionally, we have discussed upcoming stem cell-based therapeutics for several diseases which are associated with the adhesion molecules. Another major player involved in the mechanotransduction process is the nucleus and the nuclear envelope (NE), which is gaining importance as mechanosensory organelles. The Linker of Nucleoskeleton and Cytoskeleton (LINC) complexes at the NE, serves as mechanosensory hubs which connects between various cytoskeletal components of the cell (actin, microtubules and intermediate filaments) and nucleoskeletal system of the nucleus (lamins). Specifically, mutations in the lamin A, which is present at the inner nuclear membrane results in various laminopathies such as progeria (Gotzmann and Foisner, 2006), and muscular dystrophies which affects the functions of stem cell pools such as MSCs and muscle stem cells respectively (Favreau et al., 2004; Frock et al., 2006).

While several studies have highlighted the importance of these molecules and their involvement in a plethora of human diseases, there are very few studies that directly correlate mechanical signaling and disease progression. It is therefore crucial to bridge this gap in order to identify new therapeutic interventions that will increase clinical success in treating these diseases. The role of mechanotransduction in the field of regenerative medicine is starting to gain importance. Beyond regenerative medicine, the implication of microenvironmental factors that regulates both mechanical and biochemical signaling to improve the disease outcome remains largely unexplored and opens up an exciting domain of research.
